# Aggregation and Molecular Properties of β-Glucosidase Isoform II in Chayote (*Sechium edule*)

**DOI:** 10.3390/molecules25071699

**Published:** 2020-04-08

**Authors:** Alberto Cruz Rodríguez, Fabiola Anaid Sánchez Esperanza, Eduardo Pérez-Campos, María Teresa Hernández-Huerta, Laura Pérez-Campos Mayoral, Carlos Alberto Matias-Cervantes, Alexis Martínez Barras, Gabriel Mayoral-Andrade, Luis Ángel Santos Pineda, Aymara Judith Díaz Barrita, Edgar Zenteno, Carlos Romero Díaz, Ruth Martínez Cruz, Eduardo Pérez-Campos Mayoral, Edith Alhelí Bernabé Pérez, Alma Dolores Pérez Santiago, María del Socorro Pina-Canseco, Margarito Martínez Cruz

**Affiliations:** 1 Tecnológico Nacional de México/IT de Oaxaca, Oaxaca 68030, México; cruz.bio90@gmail.com (A.C.R.); plaqueta_hemostasia@yahoo.com.mx (F.A.S.E.); perezcampos@prodigy.net.mx (E.P.-C.); santospineda07@gmail.com (L.Á.S.P.); aymara.diaz@gmail.com (A.J.D.B.); edith.bp1106@gmail.com (E.A.B.P.); aperez_santiago@hotmail.com (A.D.P.S.); 2 CONACyT Facultad de Medicina y Cirugía, Universidad Autónoma Benito Juárez de Oaxaca, Oaxaca 68020, México; marte-hh28@hotmail.com (M.T.H.-H.); carloscervantes.ox@outlook.com (C.A.M.-C.); 3 Centro de Investigación Facultad de Medicina UNAM-UABJO, Facultad de Medicina y Cirugía, Universidad Autónoma “Benito Juárez” de Oaxaca, Oaxaca 68020, México; lperez.cat@uabjo.mx (L.P.-C.M.); drmayoral@gmail.com (G.M.-A.); carlos.rom.74he@gmail.com (C.R.D.); rmc.azul@gmail.com (R.M.C.); epcm@live.com.mx (E.P.-C.M.); 4 Facultad de Ingeniería, Universidad Autónoma de Querétaro, Santiago de Querétaro 76017, México; saxbarras@outlook.com; 5 Facultad de Medicina de la Universidad Nacional Autónoma de México, Ciudad de México 04510, México; ezenteno@servidor.unam.mx

**Keywords:** β-glucosidase, *Sechium edule*, aggregating protein, Cucurbitaceae

## Abstract

The presence of isoforms of β-glucosidase has been reported in some grasses such as sorghum, rice and maize. This work aims to extract and characterize isoform II in β-glucosidase from *S. edule*. A crude extract was prepared without buffer solution and adjusted to pH 4.6. Contaminating proteins were precipitated at 4 °C for 24 h. The supernatant was purified by chromatography on carboxymethyl cellulose (CMC) column, molecular exclusion on Sephacryl S-200HR, and exchange anionic on QFF column. Electrophoretic analyzes revealed a purified enzyme with aggregating molecular complex on SDS-PAGE, Native-PAGE, and AU-PAGE. Twelve peptides fragments were identified by nano liquid chromatography-tandem mass spectrometry (nano LC-ESI-MS/MS), which presented as 61% identical to *Cucurbita moschata* β-glucosidase and 55.74% identical to β-glucosidase from *Cucumis sativus*, another Cucurbitaceous member. The relative masses which contained 39% hydrophobic amino acids ranged from 982.49 to 2,781.26. The enzyme showed a specificity to β-d-glucose with a *K_m_* of 4.59 mM, a *V_max_* value of 104.3 μM∙min^−1^ and a *k_cat_* of 10,087 μM∙min^−1^ using *p*-nitrophenyl-β-D-glucopyranoside. The presence of molecular aggregates can be attributed to non-polar amino acids. This property is not mediated by a β-glucosidase aggregating factor (BGAF) as in grasses (maize and sorghum). The role of these aggregates is discussed.

## 1. Introduction

β-Glucosidases (E.C. 3.2.1.21) are enzymes that hydrolyze glycosidic bonds to release residues, non-reducing sugar terminal glycosides, oligosaccharides, and aglycones. The wide distribution of β-glucosidases in various organisms, both eukaryotes, and prokaryotes has been frequently mentioned. Their existence and high molecular preservation indicates the importance that of these molecules in the biological processes of various organisms, particularly in plants, in which a variety of functions have been reported. Among these processes participation in secondary metabolism, in the defense process [1], catabolism and lignification of the cell wall, symbiosis [2,3,4,5,6], biosynthesis and abscisic acid catabolism (ABA) [7,8], and antioxidant synthesis [9] are noted. Numerous cases of multiple enzyme isoforms have been demonstrated in plants such as sorghum, rice or maize. The function is attributed to several factors such as specificity towards one or a group of structurally related substrates, the site where it is synthesized in tissue or organelles, and the interaction with individual-specific substrates.

β-Glucosidase from *Sechium edule* [10] shows sililarities with the Cucurbitaceae family ones, particularly with those from *Cucurbita moschata* (squash) and *Cucumis sativus* (cucumber) [11]. This is indicated by the found partial sequence of amino acids that is identical to one of its fragments, as well as its specificity to the substrate. However, other partial characterization data could not be compared with different glucosidases in the family because they are not reported. Considering the classification proposed by Henrissat [12,13] which is based on the similarity between the sequence of amino acids and structure, as well as sequences of multiple-domain enzymes, we present data suggesting a clear reasoning as to why β-glucosidase in *Sechium edule* could be classified as belonging to the GH1 family. Due to a lack of information on this family, the contribution of new knowledge is considered important, as this novel enzyme could be considered for use in industrial applications in food production, energy, and pharmaceuticals, among others [14,15,16]. Its potential use depends on a more complete knowledge of its characteristics.

Previously, the purification and partial characterization of fraction I (isoform I) of β-glucosidase from chayote was performed [10]. The present study evaluates work isolation and purification, properties, and which could be classified in the GH1 family and aggregation characteristics of β-glucosidase isoform II. The juice of epicarp and mesocarp in chayote has a physiological pH 6.8 with 4.2 times more protein and 2.6 times more specific activity than in the conditions used in the extraction of isoform I [10]. Likewise, the procedure to isolate and purify isoform II has been modified, its composition, partial sequence of amino acids, identity with other members of the Cucurbitaceae family, kinetics, and the presence of molecular aggregates are shown. Finally, the possible physiological meaning of their high molecular weight molecular aggregates is discussed.

## 2. Results and Discussion

### 2.1. Preparation of Crude Extract from Sechium edule

**Figure 1:**
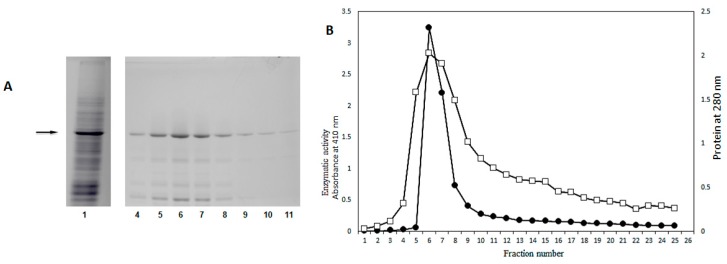
**A.** Fractions 4 to 11 with 2 mL of each, were analyzed in SDS-PAGE at 10% acrylamide, 4% bis-acrylamide. Lane 1 correspond to crude extract, the main band corresponds to β-glucosidase II, (58 kDa) note that in none of the band’s fractions molecular weight above the purified enzyme was observed. **B.** Chromatographic profile by cation exchange in CMC, column equilibrated with 200 mM sodium acetate buffer, the fractions were eluted with phosphate buffer pH 6.8. □−□ Protein, ⬤-⬤ Enzymatic activity.

The crude extract was prepared without the addition of a buffer (modified technique of Espíndola et al.) to obtain a homogenate with a physiological pH of 6.8, as in previous reports [17]. It contained 4.3 times more protein and 2.7 times more specific activity than under the extraction conditions previously reported, using sodium acetate buffer 200 mM pH 5.0. At pH 6.8, the enzyme presented the best conditions for maximum activity. In the SDS-PAGE electrophoretic analysis the *Sechium edule* β-glucosidase showed a multi-band pattern, typical of a crude extract. β-Glucosidase is recognized as an enriched band in addition to other proteins (Figure 1A Line 1). 

### 2.2. Extraction and Purification of β-Glucosidase II (Chayote Pulp Homogenate)

In the crude extract, 1566 mg of protein was obtained from 1 kg of *Sechium edule* pulp, which represents 0.16% of total protein, with 12,037 U of β-glucosidase activity. The supernatant obtained after precipitation with glacial acetic acid contained 445.36 mg of protein, corresponding to 28.59% of the protein from the initial extract with 10,479 U of enzymatic activity (Table 1). This showed an 87% recovery in one simple step, and without further manipulation, it is important to note that the specific activity was amplified three times in the extract obtained.

### 2.3. Purification of β-Glucosidase (Sechium edule) through Cation Exchange Chromatography

Figure 1B shows the chromatographic profile of the eluted protein through of cation exchange chromatography, packed with CMC, and eluded with phosphate buffer pH 6.8. From this fraction, 19.25 mg of protein was obtained with 4,276.2 U of enzymatic activity, the specific activity was amplified 29 times (Table 1). 

The analysis SDS-PAGE of this fraction shows a band with a molecular weight of 58 kDa as was reported by Espíndola et al. [10]. It did not present protein components above the main band of 58 kDa, but we observed low molecular weight bands, in particular, those in lanes 5 to 8, as shown in Figure 1B. This electrophoretic pattern is similar to that previously reported by the same authors.

At this stage of the purification process, the precipitate obtained by centrifugation with acid buffer pH 4.6 was discarded. The supernatant was loaded into the cationic column and incubated (at -18 °C) by 12 h. The procedure was implemented to evaluate the degree of protection provided by the adsorption of the enzyme by CMC at this temperature. Considering the adhesion to the ion exchanger and the denaturation of proteins by freezing and thawing, it was observed that β-glucosidase adheres in such a way. When defrosted, it does not suffer denaturation, although other proteins with the same charge in the chayote extract were selectively excluded, obtaining more purified eluate fractions with phosphate buffer pH 6.8. The protection of the enzyme on CMC is, probably, due to the presence of non-polar amino acids, which were identified in the partial sequence of the amino acids analyzed (Table 2), forming hydrophobic interactions within the quaternary structure of the enzyme. This may allow exclusion of the surrounding water, forming “cages” and thus reinforcing the ion exchange, between charges of the hydrophilic amino acids of the β-glucosidase with the resin.

In Table 2, 179 amino acids identified in the enzyme β-glucosidase with 116 kDa of molecular mass, this sequence corresponds to 35% of the total comparative sequence of β-glucosidase from *Cucumis sativus* (cucumber), containing 39% of non-polar amino acids. The composition of the enzyme is rich in alanine, tyrosine, phenylalanine, asparagine, glycine, and leucine residues, but poor in tryptophan, methionine, and glutamine residues. In addition no cysteine was detected. Of the reported amino acids 39% were non-polar and 61% polar. According to the data obtained, it is implied that the protein contains at least one-third of non-polar amino acids, as these amino acids have a strong tendency to associate with each other inside the protein promoting hydrophobic interactions [18,19]. This suggests that the protein forms compact structures in some regions, which is a phenomenon described for other β-glucosidases for which we now have evidence as being in β-glucosidase II [20]. We think this property does not allow complete enzymatic digestion by trypsin [19], suggesting that it only hydrolyzes superficial peptides of the β-glucosidase II. 

It is interesting to note that no cysteine was found, but there is likely to be some in the rest of the protein. Consequently, we assume that this amino acid is involved in the formation of disulfide bonds, which may participate in the dimerization of the protein. Hydrophobic interactions may be abundant and narrow, and this does not allow the exposure of disulfide bonds, reducing them by 2-mercaptoethanol (2-ME), and facilitating the disaggregation.

### 2.4. Purification of β-Glucosidase (Sechium edule) by Gel Filtration

**Figure 2:**
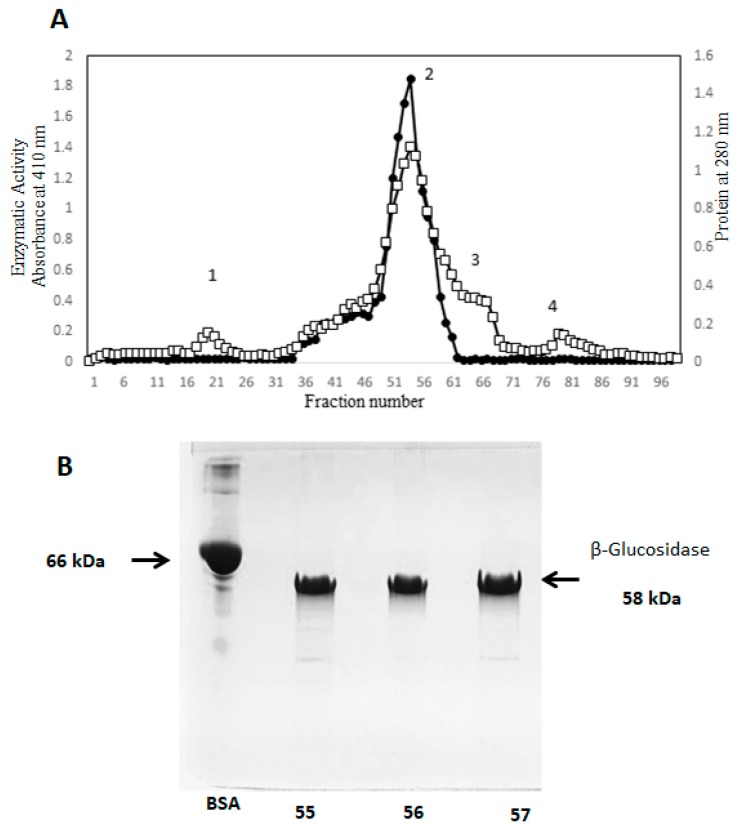
**A**. Purification chromatogram of β-glucosidase II by gel filtration, a column packed with Sephacryl S-200HR and equilibrated with phosphate buffer pH 7.2. 19 mg of protein was loaded. The active fractions obtained from the cationic column were purified, the protein was eluted with a flow of 12 mL/h., in peak 2 the purified enzyme was obtained, 2 mL fractions were collected. **B.** SDS-PAGE analysis of fractions 55, 56 and 57, low molecular weight contaminants can be seen. □−□ Protein, ⬤-⬤ Enzymatic activity.

In the fraction purified by filtration gel, four peak maxima were identified. Enzymatic activity was identifiedin the second fraction (Figure 2A) determining a total of 5.1 mg protein, with a specific activity of 803.25 U/mg/h, and an increase in purification factor of more than 100 times. Fraction 55, had higher protein concentration and higher enzymatic activity. This fraction together with fractions 56 and 57 were analyzed in dissociating electrophoresis (SDS-PAGE) at 10%. At this stage a profile with fewer bands below the band of the β-glucosidase was observed, indicating the removal of contaminant components with a molecular weight of less than 58 kDa. This allowed us to obtain a more purified protein as seen in Figure 2B. It should be appreciated in SDS-PAGE analyses, that in both previous stages of purification components of higher molecular weight to purified β-glucosidase purified were not observed (Figure 1B and Figure 2B).

### 2.5. Purification of Sechium edule β-Glucosidase by Anion Exchange Chromatography and Detection of Molecular Aggregates

**Figure 3:**
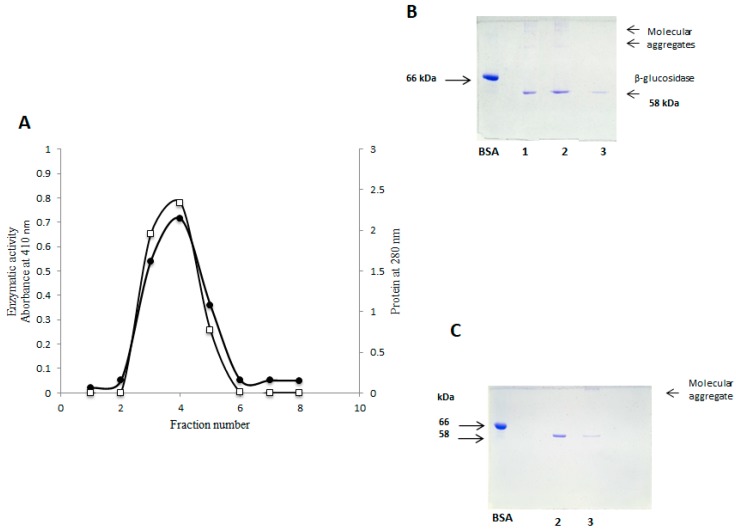
**A.** Chromatographic profile of purified β-glucosidase II by anion exchange, the QFF column (5 mL) was equilibrated with PBS 7.2. 5.1 mg of protein was loaded, this was eluted at 30 mL/h, fractions were collected of 5 mL. **B**. SDS-PAGE analysis of purified fractions 1, 2 and 3, where high molecular weight components are appreciated, which did not appear previously. **C**. SDS-PAGE of fraction 2 treated with 0.01% of 2-ME. (Line 2), fraction 2 without 2-ME (Line 3), a high molecular weight component (protein aggregates) is shown. □−□ Protein, ⬤-⬤ Enzymatic activity.

Considering that the preparations obtained by gel filtration contain “contaminant” proteins of low molecular weight (Figure 2B), we implemented an additional step for the elimination of these through the anionic exchange. Enzymatic activity was identified in the first three fractions. These were eluted with PBS pH 7.2, presenting a total protein of 4.9 mg, which represents 0.31% of the total protein (Figure 3A, Table 1) and specific activity of 841.58 U/mg/h, with an increase of 109 times. SDS-PAGE analysis shows β-glucosidase purified preparation without contaminants of lower molecular weight. However, remarkably high molecular weight components appear (Figure 3B), which were not observed in the electrophoretic analysis of the preceding stages (Figure 1A and Figure 2A), this finding suggests unmistakably high molecular weight protein remnants. These are considered molecular aggregates formed during anionic exchange chromatography.

These protein aggregates were dissolved in a PBS buffer at pH 6.8. 0.01% of 2-ME was added, it was heated for 10 min to boil and centrifuged to 10,000 rpm for 10 min, then SDS-PAGE was undergone. The electrophoretic analysis showed a pattern with a single band, corresponding to the β-glucosidase (Figure 3C, lane 2), the aggregate of high molecular weight is seen in lane 3, where the untreated sample was loaded.

### 2.6. SDS-PAGE Electrophoresis

**Figure 4:**
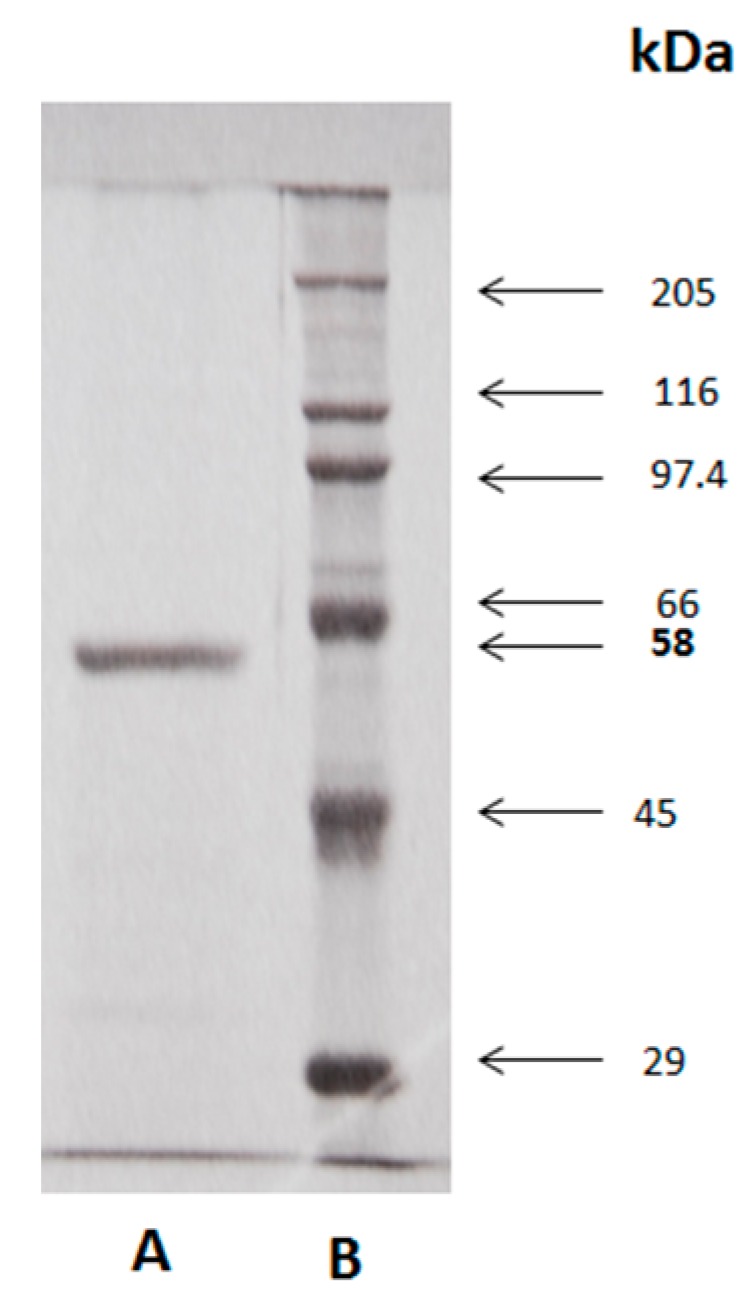
**A**. Molecular weight from purified β-glucosidase (58 kDa) from epicarp and mesocarp of *Sechium edule*, under denaturing conditions (SDS-PAGE). In 10% acrylamide gels, in the presence of SDS and 2-ME. **B**. Molecular weight standards: myosin (205 kDa), β-galactosidase (116 kDa), phosphorylase b (97.4 kDa), bovine serum albumin (BSA, 66 kDa), egg albumin (45 kDa) and carbonic anhydrase (29 kDa).

**Figure 5:**
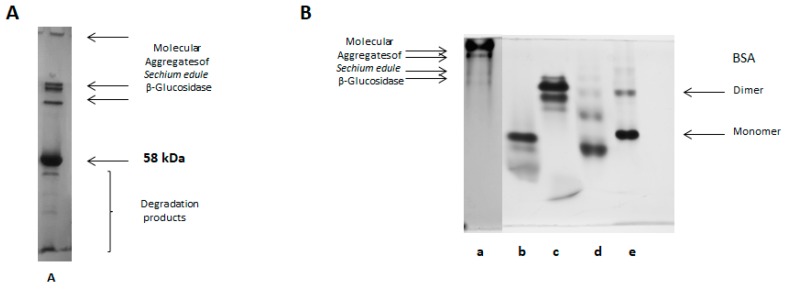

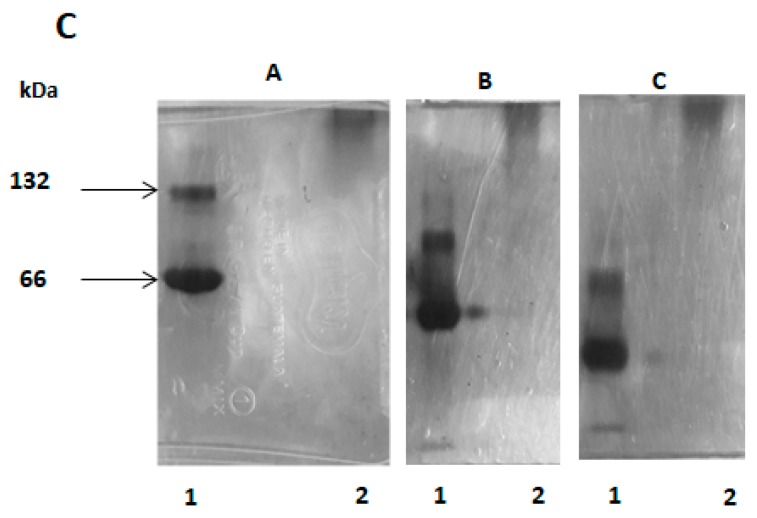
**A.** Electrophoretic analysis SDS-PAGE, note that this protein was purified and is the same sample as in Figure 3C. This protein has a marked tendency to form molecular aggregates. **B.** Electrophoretic analysis (PAGE-native) of purified β-glucosidase from *Sechium edule* after 3 weeks of storage, 10% polyacrylamide gel. Line a) 10 µg of the enzyme β-glucosidase, molecular markers, Line b) α-lactalbumin, c) carbonic anhydrase, d) egg albumin, e) BSA. **C**. Native-PAGE, of β-glucosidase II of *Sechium edule* in different concentrations of acrylamide: A) 7.5%, B) 5.5% and C) 4.5%, where 1) BSA electrophoretic mobility (monomer 66 kDa and dimer 132 kDa) and 2) β-glucosidase II from *Sechium edule*. Note the zero mobility of β-glucosidase concerning acrylamide concentration.

The homogeneity, the molecular mass of the purified β-glucosidase, and the identification of high molecular weight aggregates were evaluated using SDS-PAGE. The molecular mass of β-glucosidase isoform II was defined as a single homogeneous band with a molecular weight of 58 kDa, similar to isoform I, as shown in Figure 4. It should be noted that the analyzed protein was purified and treated with 0.01% of 2-ME, prepared at the time of analysis. The electrophoretic pattern was significantly modified, when the purified and lyophilized protein was stored for a period of 1 to 6 months. It shows a complex electrophoretic pattern, as seen in the Figure 5A. This indicates the aggregation of this β-glucosidase. 

### 2.7. Native-PAGE Electrophoresis

To confirm the formation of molecular aggregates, native-PAGE electrophoresis was also additionally used, as described in the Experimental Section. The native PAGE analysis of this isoform II clearly presents the formation of molecular aggregates on the top of the molecular markers used. More specifically, when compared to BSA with a 66 kDa monomer and a 132 kDa dimer (Sigma-Aldrich technical bulletin) as shown in Figure 5. A component of high molecular weight and the presence of other proteins of lower weight inside of the gel, which is indicative of non-interaction with acrylamide and reinforces the hypothesis of aggregate formation (Figure 5B, line a). The aggregation phenomenon becomes more evident when a lyophilized 6 months-storage sample is dissolved in phosphate buffer pH 7.2 and analyzed, no-defined bands pattern were observed, instead, a smeared sample was observed in the top of the separating gel, even opening the gel pore from 7.5 to 4.5% acrylamide concentration, which is evidenced by BSA protein used as a control, as shown in Figure 5C.

### 2.8. Electrophoretic Analysis in Acetate-Urea pH 4.4

**Figure 6:**
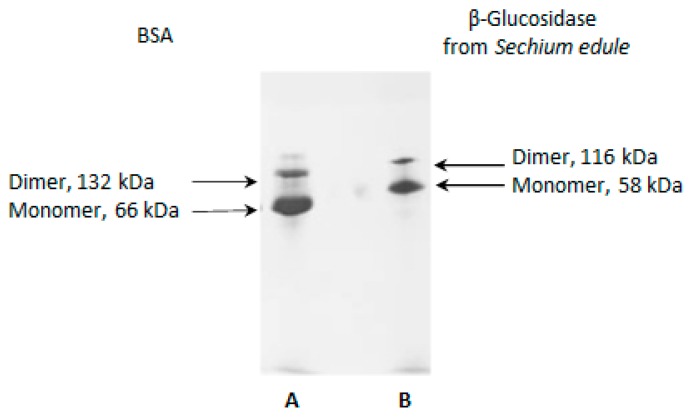
Electrophoretic analysis in Acetate-Urea (AU-PAGE) at pH 4.4 in 10% acrylamide, where lane **A** is BSA and lane **B** correspond to β-glucosidase from *Sechium edule*. In this electrophoresis the protein migrates as a cation.

Molecular aggregates of β-glucosidase were subjected to acid electrophoresis. In Figure 6, line B, we observed two bands in the separating gel, which have less migration towards the anode than BSA (Figure 6, line A), as in the reference protein. This data describes β-glucosidase as a more acidic protein than BSA. The bands mentioned correspond to its native weight of 116 kDa (dimer) and a band of 58 kDa (monomer). We consider that the electrophoretic pattern of BSA in the same condition of analysis (Figure 6, line A) is similar to that observed in PAGE-native, in which charge isomers were identified. The pattern is similar to that reported in the BSA technical bulletin, already mentioned, and as shown in the Figure 5B, lane e. As in PAGE-native, β-glucosidase II was identified as a molecular aggregate and is not defined as a band (Figure 5B).

### 2.9. Analysis in Two-Dimensional Electrophoresis

**Figure 7:**
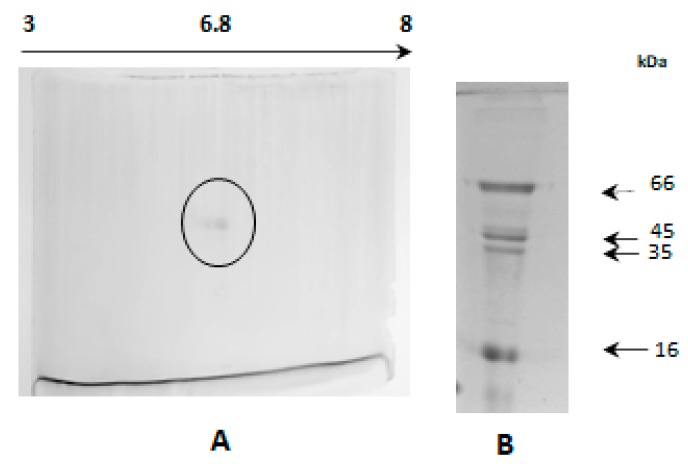
Two-dimensional (2D) electrophoresis of fresh, purified from β-glucosidase from *Sechium edule* II. Where: **A**. 6.8 pI and **B**. Molecular weight markers: BSA (66 kDa), ovalbumin (45 kDa), pepsin (35 kDa) and trypsin inhibitor (16 kDa).

The protein analyzed in 2D was appreciated as a spot, indicating a molecular mass of 58 kDa with a pI range of 6.8 and corresponding to the isoform II of β-glucosidase (Figure 7). This allows us to confirm the data obtained by AU-PAGE and defines it as an isoform (II) because of not additional spots (proteins).

### 2.10. Specificity Assays with Artificial Substrates and Kinetic Parameters 

The results obtained shown that specificity substrate for this enzyme was β-d-glucose ˃ β-d-galactose ˃ β-d-fucose, using the general substrate *p*-nitrophenyl (*p*-NP) incorporated to these glucosides. This proves that β-glucosidase isoform II has a recognizable, non-specific feature (Table 3), presenting specificity in a narrow margin of related substrates of the same group. This result is similar to that described in other β-glucosidases [21].

**Figure 8:**
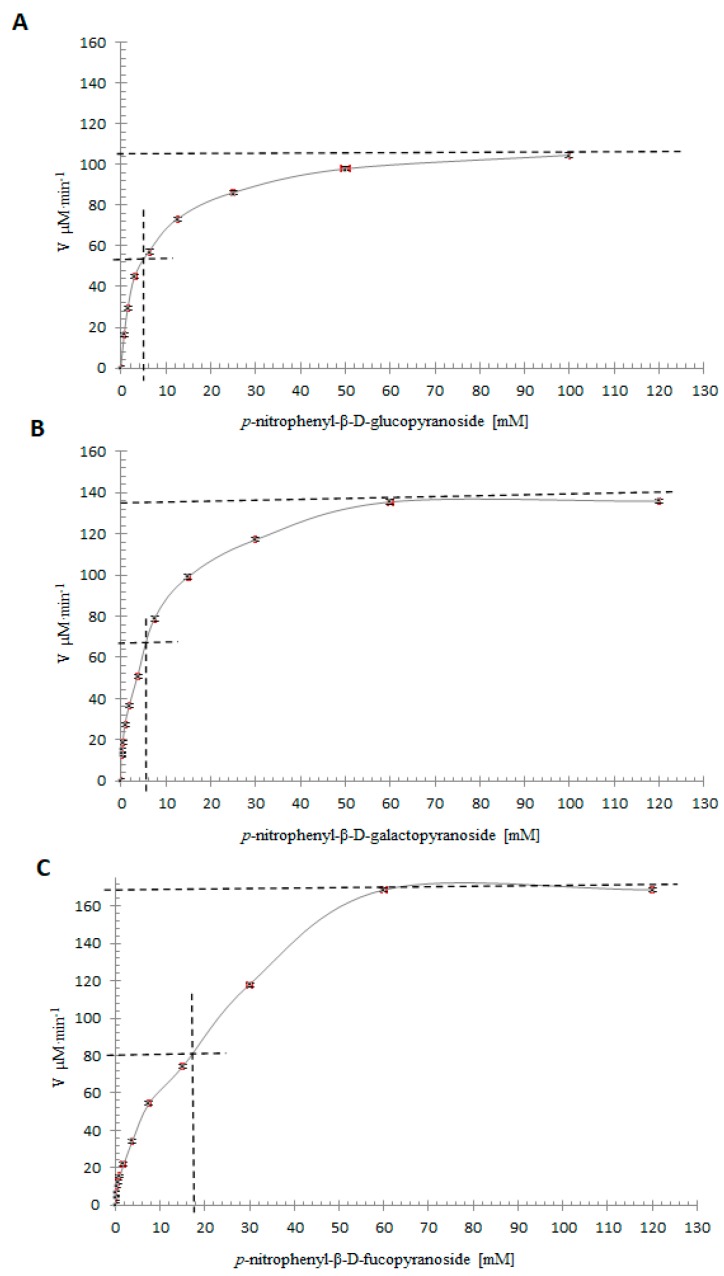
**A.** Enzymatic kinetics of β-glucosidase isoform II from *Sechium edule.* 4 μg of the purified enzyme with different concentrations of *p*-nitrophenyl-β-d-glucopyranoside, obtained a *K_m_* of 4.6 mM and *V_max_* = 104.5 mM∙min^-1^. **B.** Enzymatic kinetics of β-glucosidase isoform II from *Sechium edule*, 4 μg of the purified enzyme with different concentrations of *p*-nitrophenyl-β-d-galactopyranoside obtained a *K_m_* of 5.6 mM and *V_max_* = 135 mM∙min^-1^. **C.** Enzymatic kinetics of β-glucosidase isoform II from *Sechium edule*, 4 mg of the purified enzyme with different concentrations of *p*-nitrophenyl-β-d-fucopyranoside, a *K_m_* of 15.95 mM was obtained and *V_max_* = 168.3 mM∙min^-1^.

*K_m_* was determined as a measure of the affinity of the enzyme for its substrate, therefore it depends on the type of substrate, among other variables, such as is reported for other β-glucosidases [10,18]. The kinetic parameters show slightly different values than those reported previously, with *p*-NP-β-d-glucopyranoside, *p*-NP-β-d-galactose and *p*-NP-β-d-fucopyranoside. In Table 4, we present values of *K_m_* (mM), *V_max_* (μM∙min^−1^∙mg^−1^), *k_cat_* (min^−1^), and the catalytic efficiencies for β-glucosidase II by *k_cat_*/*K_m_* ratios (Figure 8).

Activity was assayed using various concentrations of substrates, at pH 5.5 and at 40 °C, with 4 μg of β-glucosidase. The release of *p*NP was measured, as described in the experimental methods. The kinetic constants were determined by analyzing the data nonlinear regression with Prism 6 (GraphPad Software. Inc., San Diego, CA, USA). All values are the averages of two separates experiments done in triplicate.

### 2.11. Analysis of Tryptic Peptides 

While looking for sequence similarities with other proteins, several Cucurbitaceae β-glucosidases were identified, according to the Basic Local Alignment Search Tool (BLAST). It showed an identity of 60.89% and a value of 2e-59 in cyanogenic β-glucosidase-like (*Cucurbita moschata*) (XP 022960706.1); in cyano β-glucosidase-like (*Cucurbita pepo* subsp. *Pepo*) (XP 023516390.1) with 60.34% identical and a value of 9e-59. It also predicted cyanogenic β-glucosidase-like (*Cucumis melo*)(XP 008450457.1) with 56.22%, and 2e-57; and predicted a cyanogenic β-glucosidase-like (*Cucumis sativus*) (XP 011660112.1) with 55.74% identity and a value of 5e-57. The identity of these sequences corresponds to the primary structure of these proteins. The β-glucosidase II showed high identity with four glucosidases of the Cucurbitaceae family, ranging from 56% in *Cucumis sativus* to 61% in *Cucurbita moschata*.

**Figure 9:**
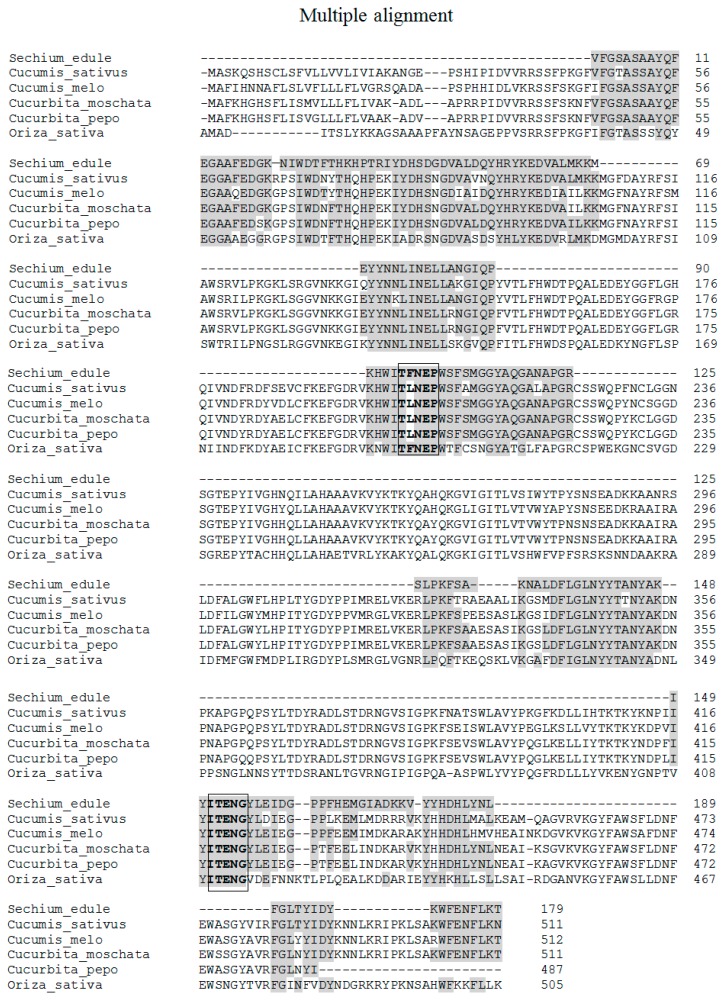
Partial sequence identity deduced by β-glucosidase isoform II from *Sechium edule, Cucumis sativa, Cucurbita moschata, Cucurbita pepo, Cucumis melo*. The multiple sequences were aligned using BLAST, in this figure the regions of identity with other reported β-glucosidases of some members of the *Cucurbitaceae* and *Oriza sativa* (*Gramineae*) families are indicated. There are also two motif peptides (in the boxes) showing those involved in the recognition of highly conserved sugar and the catalytic site in the GH1 family.

The sequence obtained corresponds to 35% of both glucosidases (179 out of 511 amino acids). Additionally, the highest reported identity corresponds to *Cucurbita moschata* as mentioned above. The molecular mass obtained in *Sechium edule* is similar to *Cucumis sativus* and *Cucurbita moschata*, 58.142 kDa, with an identical number of amino acid residues (Table 5 and Figure 9).

**Figure 10:**
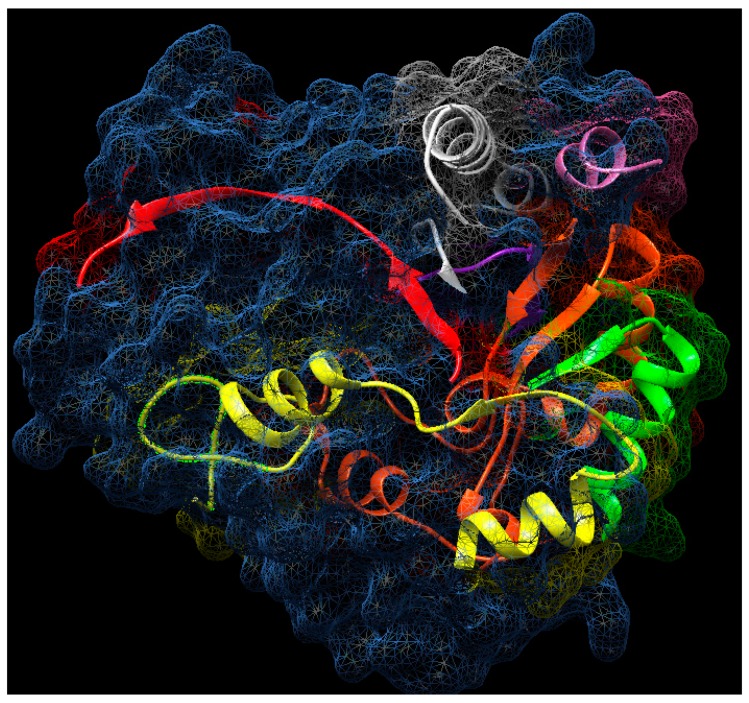
Location of β-glucosidase isoform II from *Sechium edule* amino acids residues (in cartoon colors) using the tertiary structure of β-glucosidase from rice as a template (surface representation in dots and spirals). 70% of the sequenced residues of β-glucosidase from *Sechium edule* are found on the surface of the protein, except residues 173 to 196 (yellow), residues 316 to 329 (red), and residues 456 to 462 (purple). 3PTM.pdb modified, using Chimera [23].

In the terminal amino region, four homologous peptides were identified with *Cucurbita moschata* β-glucosidase. The VFGSASAAYQFEGAAFEDGK peptide from residue 46 to 65 with 100% identity, the NIWDTFTHKHPTR peptide from residue 68 to 80 with a 62% identity, the IYDHSDGDVALDQYHR peptide with 94% identity, from residue 81 to 96, and the peptide YKEDVALMKK with 90% identity, from residues from 97 to 106 (Table 5). With respect to residues 46 to 106, a continuous polypeptide of 59 amino acids was identified (Figure 9), which presents an identity of 79% with the tertiary structure of *Oryza sativa* (rice) in accordance with the I-TASSER server [22]. Also, when that there were no reports in databases of tertiary structures of β-glucosidases in *Cucurbitacea*, an *Oryza sativa* structure was used to identify the possible spatial location of the polypeptide obtained from *Sechium edule*. It was found that this sequence is on the surface (Figure 10), and suggests that peptides, obtained by digestion with trypsin, are located on the surface. Therefore, it is likely that the presence of non-polar amino acids (Table 2), are found inside the protein establishing strong hydrophobic interactions. Consequently, this forms molecular aggregates, which could have contributed to obtain a greater number of sequenced peptides.

Other homologous sequences located in the middle region of the protein were: the EYYNNLINELLANGIQP peptide from residue 137 to 153 that showed 94% identity; the HWITFNEPWSFSMGGYAQGANAPGR peptide from residue 199 to 223 with 96% identity (Table 5), which contains a conserved region (highlighted in the reframe) (Figure 9) that participates in the recognition of sugar, and is located in the catalytic site. This is conserved in families of β-glucosidases and classified into glycosyl hydrolases 1 (GH1) family. This motif peptide, in some members of the reported Cucurbitaceae family, presents the replacement of phenylalanine (F) by leucine (L) residues (Figure 9). It could be like that the recognition of the catalytic site is similar because of the since this substitution is exchanged by an amino acid of the same group. The sequence identified in β-glucosidase II is similar to that reported in several grasses [24,25,26,27], such as maize, sorghum, oat, and rice. Other peptides in the middle portion of the sequence, were SLPKFSAK, from residue 324 to 332 with 75% identity, NALDFLGLNYYTANYAK from residue 338–354 with 88% identity, and the IYITENGYLEIDGPPFHEMGIADK sequence of residue from 416–439 with 62.5% identity. These sequences were identical to β-glucosidase from *Cucurbita moschata*. In this last peptide, a second motif sequence (highlighted in the reframe) was found identical to those found in other Cucurbitaceae (Figure 9), and grasses [22,24,25,26,27]. These sequences are highly conserved, therefore, the similar three-dimensional structure of the eight-stranded β/α barrel (TIM-barrel) is possible, allowing classification with β-glucosidase II in the GH1 family [21]

At the end of the carboxyl-terminal sequences, another 3 peptide sequences were identified: KVYYHDHLYNLR from residue 440–451 with 67% identity, FGLTYIDYK from residue 483 to 491 with 100% identity, and WFENFLKT from residue 504–511, also with 100% identity. We obtained 35% of the total sequence concerning *Cucurbita moschata* and *Cucumis sativus*. These data contain a similar number of amino acids and molecular masses to β-glucosidase II in *Sechium edule* (Table 5). It should be remembered that the highest peptide similarity was 61% identity with *Cucurbita moschata*.

### 2.12. Molecular Aggregates

We can conclude that the components of high molecular weight are the molecular aggregates identified in isoform II, and not those present in isoform I. In the electrophoretic analysis of purified fractions by cation exchange and gel filtration (Figure 2A), no molecular weight compounds greater than β-glucosidase and BSA were observed. These compounds were found in fractions obtained by anionic exchange. The aggregate was dissolved in 50 mM citrate buffer pH 6.8 with 0.01% of 2-ME. In line 2, Figure 3C, the absence of molecular aggregates of the β-glucosidase after the above-mentioned treatment, is observed. This is corroborated in line 3 by their presence in the same sample under standard conditions of SDS-PAGE in view of the high molecular weight component, at the top of the separating gel. This property is common in members GH1 family of β-glucosidase, particularly, in isoforms such as β-glucosidases from *Zea mays* (ZmGlu1) and *Sinapis alba* (MA1 myrosinase), which are also involved in the defense of plants in vivo [19].

One of the factors that could contribute to the formation of molecular aggregates is the presence of non-polar amino acids, in which case, β-glucosidase II has a proportion of 30%, with respect to the sequence obtained (Table 2). An example of molecular aggregates is found in maize with β-glucosidase aggregating factor (BGAF) [28,29], which also presents polyspecific lectin activity, although in was not identified in preparations of crude extracts and purified fractions of *Sechium edule*, previously described in exudates of the fruit [30]. Recent studies suggest that the BGAF does not protect β-glucosidase in the case of maize. Further studies are needed [19].

The physicochemical properties described in β-glucosidase II, which could contribute to their stability are not completely understood, however, the common structural characteristics reported suggest that they are responsible for their stableness. This condition allows β-glucosidase to operate under special conditions, as the intestinal tract in certain insects. Vassão et al. describes the digestion resistance of three β-glucosidases in *Zea mays* and BGAF associated with this enzyme, myrosinase from *Sinapis alba*, and the β-glucosidase from *Prunus dulcis* (almonds). These proteins showed high resistance to the proteolytic enzymes from predator intestine (*Spodoptera littoralis*), maintaining the ability to catalyze the hydrolysis of glucosides [19]. However, there is no consensus on the belief that protein aggregates confer protection on the β-glucosidase. This needs for further study.

There is a lack of information about the formation of these aggregates in the β-glucosidase II in from *Sechium edule.* In fact, it is a little-known property. In this work, we report on the presence of non-polar amino acids (Table 2) which could participate in the formation of hydrophobic interactions, and point to β-glucosidase II as a pore-forming protein of molecular aggregates. This is possible due to the participation of at least one disulfide bond that would contributes to reinforcing this phenomenon, although the presence of cysteines was not identified. To support this hypothesis, the sequences obtained from BLAST (Figure 9) by multiple alignments with several β-glucosidases of the Cucurbitaceae and Gramineae families were compared, and the existence of four cysteines was identified. The presence of at least one disulfide bond, in the sequence of β-glucosidase II, may be involved in the dimerization of this enzyme, by understanding native molecular mass of 116 kDa, and monomers of 56 kDa. Its treatment with 2-ME disaggregated the protein, as shown in SDS-PAGE (Figure 3C) contributing to the reduction of disulfide bonds.

The aggregation phenomenon in these Cucurbitaceae spp. is attributed to the intrinsic nature of this protein and, therefore, to the presence of hydrophobic amino acids in the tertiary structure. The biological significance of the formation or existence of molecular aggregates may be related to an evolutionary plant strategy to protect them from the degradation of the phytophagous insect by endogenous enzymes. It is accepted that one of the main functions of β-glucosidases is their participation in chemical defense against predatory insects, by continuously releasing toxic aglycones [19].

It is understood that the presence of non-polar amino acids is strongly associated with aggregates in the folded protein or core of proteins. As the molecules gather they have to form aggregates in aqueous solutions, regard water entropy, which increase when water molecules are released during protein folding in large aggregate formation. In β-glucosidase II, we should consider that its water content is 90%, therefore, the protein is in the ideal medium for the formation of the active aggregates.

However, it is not clear whether the molecular aggregation of this class of protein has a protective effect against degradation by proteolytic enzymes. We suggest that due to its hydrophobic nature (similar to β-glucosidase 1 from maize, myrosinase, and almond), it does play an important role in protection. However, more studies should be carried out in this direction.

The study of this property will help us to understand the physiological role of molecular aggregates and will be of great interest (given the limited data) in other plants because of its high economic value, such as in the cultivation of *Sechium edule,* where some groups of bed bugs of the suborder Hemiptera [31] have been identified. At present, it is difficult to establish the role of these insects, however, some may potentially to become a pest for crops and the production of *Sechium edule*. Therefore, it is important to pursue the study of β-glucosidase and the possible role of molecular aggregates could have in the protecting this protein and its possible participation in the defense mechanisms in Cucurbitaceae species.

## 3. Experimental 

### 3.1. Preparation of the Modified Crude Extract of Sechium Edule

To obtain β-glucosidase II, the method reported by Espíndola et al. [10] was used with the modifications described below. Chayote (*Sechium edule*) was washed and peeled. One kg of pulp (epicarp and mesocarp) was weighed and macerated without adding any buffer. The crude extract was filtered and centrifuged at 10,000 rpm for 60 min at 4 °C. The supernatant was adjusted to pH 4.6 with glacial acetic acid and incubated at 4 °C for 24 h. The precipitated was discarded and the supernatant was quantified [32] using BSA as standard and *p*NPG as an enzymatic activity control [33].

### 3.2. Enzyme Purification

#### 3.2.1. Cation Exchange Chromatography

Protein (445.36 mg) displaying 10,479 U of enzymatic activity, was loaded into a cation exchange column (20 cm in length × 1 cm in diameter) packed with CMC. Prior to this, it was equilibrated with sodium acetate (NaAc) buffer 200 mM pH 5.0 to obtain at ≤ 0.01 to 280 nm optical density, at room temperature (25 °C ± 0.5). Once the sample was loaded, the column was incubated at −18 °C for 18 h. After this period, the column was washed with the same buffer at room temperature. The enzyme was eluted with 200 mM phosphate buffer pH 6.8 with a flow of 35 mL/h. Fractions of 2.0 mL were collected, protein and enzymatic activities were quantified as described above. 

#### 3.2.2. Molecular Exclusion Chromatography

Fractions, with the highest enzymatic activity, obtained from the cationic exchange chromatography, were concentrated and loaded. A fraction with 19.25 mg of protein with 4279 U of enzymatic activity was eluded through a gel filtration column of 100 cm and length x 1.5 cm diameter, packed with Sephacryl S-200HR (Sigma-Aldrich, Saint Louis, MO, USA), equilibrated with PBS 200 mM pH 7.2 with a flow of 10 mL/h, at room temperature. Fractions of 2.0 mL were collected, which were quantified protein and enzymatic activity.

#### 3.2.3. Anionic Exchange Chromatography

4,102 U obtained from gel filtration with 5107 mg of protein were eluted through a commercial 5 cm long × 1.5 cm in diameter Hi Trap Q Sepharose Fast Flow (QFF) anion exchange column (Sigma Chemical, Co., Saint Louis, MO, USA). This was previously equilibrated with 200 mM PBS pH 7.2 with a flow of 30 mL/h at room temperature, collecting 5.0 mL fractions. Protein and enzymatic activity were quantified.

#### 3.2.4. Dialysis and Lyophilization

The fractions with the highest enzymatic activity obtained from anion exchange chromatography were dialyzed against triple distilled water at 4 °C with several changes for 48 h and lyophilized for 24 h, using dialysis bags with pores of maximum diameters for proteins of 13,000 kDa (Sigma Chemical, Co.).

### 3.3. Determination of Molecular Mass and Identification of Molecular Aggregates in SDS-PAGE

Homogeneity, molecular mass, and identification of high molecular weight aggregates of β-glucosidase II purified from *Sechium edule* were evaluated in electrophoresis SDS-PAGE according to Laemmli [34].

#### 3.3.1. Native-PAGE Electrophoresis

To identify the formation of molecular aggregates, native-PAGE electrophoresis was employed using 0.05 M phosphate buffer pH 8.3, according to Hedricks and Smith [35], and Ferguson et al. [36].

#### 3.3.2. Acetate-Urea Acid Electrophoresis

β-Glucosidase previously identified as a molecular complex was analyzed by denaturing acid electrophoresis in AU-PAGE using the method described by Reisfeld et al. [37]. In the running buffer, 1.56% β-alanine and 4% glacial acetic acid pH 4.4, were used, and pyronin was added as an electrophoretic mobility indicator. The enzyme migrated like a cation. 

#### 3.3.3. Two-Dimensional Electrophoresis

A 2D electrophoresis analysis of the purified enzyme was performed, following the methodology described by O’Farrell et al. [38]. In all cases, the gels were stained with 0.1% Coomassie brilliant blue G-250 (Sigma Chemical, Co).

### 3.4. Determination of Partial Amino Acid Sequence Using Nano-LC-ESI-MS/MS

The enzyme was previously reduced with dithiothreitol (DTT) (Sigma-Aldrich), alkylated with iodoacetamide (Sigma-Aldrich) and digested “in solution” with trypsin (Promega Sequencing Grade Modified Trypsin; Madison, WI, USA), in 50 mM ammonium bicarbonate pH 8.2, and incubated for 18 h at 37 °C. The peptides produced by enzymatic cleavage were desalted with Zip Tip C18 (Millipore; Billerica, MA, USA) and applied in an liquid chromatography-mass spectrometry with a nano-electrospray ionization source system (Nano-LC-ESI-MS/MS) (Quadrupole/time of flight, Ultima API, Micromass, Manchester, UK) composed of an EASY-nLC II nanoflow pump (Thermo-Fisher Co.; San Jose, CA, USA) coupled to a LTQ-Orbitrap Velos mass spectrometer (Thermo-Fisher Co.).

Calibration was performed with a Calmix solution (*N*-butylamine, caffeine, Met-Arg-Phe-Ala peptide and Ultramark 1621). In order to calibrate the LTQ Velos with ion trap (IT) module and the Orbitrap module with FT (Fourier Transform) mass detector in ESI positive ionization mode. *N*-Butylamine (73.14 Da) is included to extend mass calibration to lower values of *m*/*z* of 5 parts per million (ppm).

In the nano-flow liquid chromatography, a gradient system of 10–80% solvent B (water/acetonitrile with 0.1% formic acid) and solvent A (water with 0.1% formic acid) was used for 120 min using a capillary column (ID 0.75 µm and 10 cm length RP-C18). The flow of the LC system was 300 nanoliters/minute.

Total ion scanning (Full Scan) was performed on the Orbitrap analyzer with a resolution power of mass (RP Power; RP = m/FWHM) of 60,000 kDa. Peptide fragmentation was performed using the methods of Collision-Induced Dissociation (CID) and High-Energy Collision Dissociation (HCD). All spectra were acquired in positive detection mode. The execution and capture of the fragmentation data was performed depending on the total ion scan according to the pre-determined charges (only ions with a z2+, z3+ and z4+ charge). These were fragmented with an isolation width of 2.0 (*m*/*z*), normalized collision energy of 35 arbitrary units, Q activation of 0.250, activation time of 10 milliseconds and maximum injection time of 10 milliseconds per micro-scan. During the automatic data capture, the dynamic ion exclusion was used: (i) 200 ion exclusion list, (ii) 30 seconds (s) pre-exclusion time, and (iii) 70 s exclusion time [39].

### 3.5. Bioinformatic Analysis

Spectrometric data was obtained in raw format in the Proteome Discoverer 1.4 program (Thermo-Fisher Co.) through the Sequest HT search engine. For the identity search, we used the database of all proteins organisms, *Sechium*, and β-glucosidase (UniProt). An FDR-False Discovery Rate of 0.01 (Minimum) and FDR of 0.05 (Maximum) was used in addition to the inverted database (Decoy database) as a tool of the “Percolator” validation program. The maximum tolerance of molecular mass difference of the precursor ion and the theoretical versus experimental values (precursor mass tolerance) were compared, it was 20 ppm and the tolerance for the fragments obtained by dissociation of the precursor ion (fragment mass tolerance) of 0.6. For the automatic search, constant modifications (carbamide-methylation of cysteines) and variables such as oxidation of methionine (M) and deamination of asparagine (N) and glutamine (Q) were established.

After the automatic search, manual sequencing of the MS/MS spectra was performed. The sequences obtained are reported in Table 1, with their ion *m*/*z*, z^+^ (charge), molecular mass (MW), and the sequence identity was obtained using BLAST [40].

### 3.6. Enzymatic Activity

Enzymatic activity of the crude extract and all purified fractions was measured using *p*NPG as a substrate. The fractions were diluted (1:100) in 0.2 M sodium citrate pH 5.0. 50 μL of the sample was incubated with 50 μL of the 5 mM substrate at 25 °C for 5 min in a 96 well microtiter plate. The reaction was stopped with 50 μL of 400 mM sodium bicarbonate (NaHCO_3_). The *p*NP produced was quantified at 410 nm, one unit of activity was defined as the amount of enzyme capable of catalyzing the hydrolysis of a 1 μM of substrate per hour. Specific activity (SA) was expressed in units of enzyme per mg of protein [29,33]. Protein concentration was determined through the Bradford method [32].

### 3.7. Determination of Kinetic Parameters

*K_m_*, *k_cat_*, and the *K_m_/k_cat_* ratio were determined using 0–100 mM of *p*-nitrophenyl-β-d-glucopyranoside, *p*-nitrophenyl-β-D-galactopyranoside and *p*-nitrophenyl-β-D-fucopyranoside by each 4 μg of β-glucosidase from *Sechium edule* in 0.2 M sodium citrate buffer pH 5.0, in 100 μL reaction volume at 40 °C for 5 min. The data obtained were analyzed using nonlinear regression with Prism 6 (GraphPad Software. Inc., San Diego, CA, USA).

Additionally, the following substrates were also evaluated to determine the specificity of β-glucosidase II using the same methodology: *p*-nitrophenyl-β-D-cellobioside, *p*-nitrophenyl-β-D-mannopyranoside, *p*-nitrophenyl-β-D-lactopyranoside, *p*-nitrophenyl-β-D-maltopyranoside, *p*-nitro-phenyl-β-D-*N*-*N*’-diacetylchitotriose, *p*-nitrophenyl-β-D-*N*-acetylgalactosamine, and *p*-nitrophenyl-α-D-glucopyranoside. We only report *p*-nitrophenyl-β-D-glucopyranoside, *p*-nitrophenyl-β-D-galactopyranoside, and *p*-nitrophenyl-β-D-fucopyranoside results.

## 4. Conclusions

The process of purification of β-glucosidase from *Sechium edule* was adapted, incorporating other elements described and discussed above. We obtained a homogeneous purified preparation of the isoform II, which forms molecular complexes of high molecular weight, with aggregating properties. These complexes were not found in previous studies of isoform I [10]. We attribute this property to the content of non-polar amino acids that form hydrophobic, intra and intermolecular interactions. In contrast to β-glucosidase from Gramineae, which has previously been reported as responsible for the formation of molecular aggregates into the protein. β-Glucosidase aggregating factor, is not present in *Sechium edule* described here. The partial sequence of 179 amino acids corresponds to 35% of the β-glucosidase sequence of cyanogenic β-glucosidase-like (*Cucurbita moschata*), a member of the Cucurbitaceae family. The sequence reported in the Fasta format of β-glucosidase II was analyzed by BLAST (https://blast.ncbi.nlm.nih.gov/Blast.cgi#alnHdr_XP_022960706.1), obtaining an identity of 61% with *Cucurbita moschata* with an e-value of 2e-59.

Two motif peptides TFNEP and ITENG, were identified as belonging to the catalytic site that contributes to the recognition of the substrate. This has a sequence similar to that reported in grasses and legumes which are identical to those reported in both Cucurbitaceae, and Gramineae. Their specificity was directed towards β-d-glucose ˃ β-d-galactose ˃ β-d-fucose and their kinetic parameters were similar between these substrates. The characteristics described in this work suggest the identity of β-glucosidase II as belonging to the GH1 family.

## Figures and Tables

**Table 1:** β-Glucosidase activity during the purification process of *Sechium edule* from 1 kg of pulp homogenate (chayote juice).

Fraction	Total Protein (mg)	Activity of β-Glucosidase (U)	Specific Activity (U/mg)	Purification Factor	Yield (%)
Crude pulp extract	1565.58	12,037.56	7.69	1	100
Glacial acetic acid Protein supernatant	445.36	10,479.30	23.53	3.05	87.05
Cationic chromatography CMC	19.25	4276.20	222.14	28.88	36
Gel filtration (S-200HR)	5.107	4102.20	803.25	104.45	34
Anionic chromatography (QFF)	4.855	4085.90	841.58	109.36	33.90

**Table 2:** Amino acid composition of the of β-glucosidase isoform II of *Sechium edule.*

Amino Acids		Res/mol	Residues/100%
Val	V	4	2.23
Phe	F	12	6.70
Gly	G	14	7.82
Ser	S	7	3.91
Ala	A	16	8.94
Tyr	Y	17	9.49
Gln	Q	4	2.23
Glu	E	10	5.58
Asp	D	12	6.70
Lys	K	12	6.70
Asn	N	13	7.26
Ile	I	10	5.58
Trp	W	4	2.23
Thr	T	8	4.46
His	H	8	4.46
Pro	P	7	3.91
Arg	R	4	2.23
Leu	L	14	7.82
Met	M	3	1.67
Totals		179	100

**Table 3:** Substrates to determine the specificity of β-glucosidase isoform II from *Sechium edule.*

**Artificial Chromogenic Specific Substrates**
*p*-Nitrophenyl-β-d-glucopyranoside
*p*-Nitrophenyl-β-d-galactopyranoside
*p*-Nitrophenyl-β-d-fucopyranoside
**Non-Specific Substrates**
*p*-Nitrophenyl-β-d-manopyranoside
*p*-Nitrophenyl-β-d-lactopyranoside
*p*-Nitrophenyl-β-d-maltopyranoside
*p*-Nitrophenyl-α-d-glucopyranoside
*p*-Nitrophenyl-β-d-*N*,*N*′-diacetylcytotriose
*p*-Nitrophenyl-β-d-cellobioside
*p*-Nitrophenyl-β-d-*N*-acetylgalactosamine

**Table 4:** Kinetic constant of β-glucosidase isoform II from *Sechium edule.*

Substrate	*K_m_* (mM)	Standard Error *K_m_*	*k*_cat_ (min^−1^)	Standard Error *kcat*	10^−3^ × *k*_cat_/*K_m_* (mM^−1^ min^−1^)
*p*NPGlc	4.59	0.49	10,086	1008	2197
*p*NPGal	5.72	0.45	13,718	1070	2398
*p*NPFuc	16.11	2.62	16,289	2638	1011

**Table 5:** Analysis of tryptic peptides from the 58 kDa subunit of β-glucosidase isoform II from *Sechium edule* (Sebg) by nano-LC-ESI-MS/MS.

Name	Ion	*m*/*z*^1^	MW ^2^ (Da)	Sequence	Start-End Sequence % Identical
Sebg1	1129.01	2	2256.02	VFGSASAAYQFEGAAFEDGK	46-65-Cmbg 100% y Csbg 85%
Sebg2	903.42	3	2702.26	NIWDTFTHKHPTR	68-80-Cmbg 62% y Csbg 54%
Sebg3	942.53	2	1883.06	IYDHSDGDVALDQYHR	81-96-Cmbg 94% y Csbg 81.25%
Sebg4	408.89	3	1223.67	YKEDVALMKK	97-106-Cmbg 90% y Csbg 80%
Sebg5	122.63	2	2439.26	EYYNNLINELLANGIQP	137-153-Cmbg 94% y Csbg 88.23%
Sebg6	1391.63	2	2781.26	HWITFNEPWSFSMGGYAQGANAPGR	199-223-Cmbg 96% y Csbg 88%
Sebg7	538.82	2	1075.64	SLPKFSAK	324-332-Cmbg 75% y Csbg 50%
Sebg8	1164.07	2	2326.14	NALDFLGLNYYTANYAK	338-354-Cmbg 88% y Csbg 76%
Sebg9	1333.14	2	2664.28	IYITENGYLEIDGPPFHEMGIADK	416-439-Cmbg 62.5% y Csbg 62.5%
Sebg10	811.92	2	1621.84	KV-YYHDHLYNLR	440-451- Cmbg 66.6% y Csbg 50%
Sebg11	560.29	2	1118.58	FGLTYIDYK	483-491-Cmbg 100% y Csbg100%
Sebg12	542.57	2	982.49	WFENFLKT	504-511-Cmbg 100% y Csbg 87.5%

^1^ Ionic charge of the peptide. ^2^ Molecular weight. Peptide sequence position to *Cucumis sativus* (cucumber) and *Cucurbita moschata* β-glucosidases (Csbg) (pumpkin).
